# Geographic Variation in the Prevalence of *Candidatus* Neoehrlichia procyonis in Raccoons (*Procyon lotor*) in the United States and Canada

**DOI:** 10.1002/mbo3.70017

**Published:** 2025-04-07

**Authors:** Meghan Lewis, Kayla B. Garrett, Christopher A. Cleveland, Sonia M. Hernandez, Mark Swain, Michael J. Yabsley

**Affiliations:** ^1^ Southeastern Cooperative Wildlife Disease Study, Department of Population Health, College of Veterinary Medicine University of Georgia Athens Georgia USA; ^2^ Warnell School of Forestry and Natural Resources University of Georgia Athens Georgia USA; ^3^ Center for Ecology of Infectious Diseases University of Georgia Athens Georgia USA

**Keywords:** procyonid, surveillance, tick‐borne pathogen, wildlife

## Abstract

Raccoons (*Procyon lotor*) are reservoirs for pathogens of other wildlife species, domestic animals, and humans, including several tick‐borne pathogens. A relatively understudied organism in raccoons is *Candidatus* Neoehrlichia procyonis which has been detected in raccoons from the southeastern United States. A related species in Europe and Asia, *Neoehrlichia mikurensis*, uses rodents as reservoirs and *Ixodes* spp. as vectors; however, studies on rodents suggest they are not susceptible to *Ca*. N. procyonis. *N. mikurensis* has been associated with cases of neoehrlichiosis in people and dogs, which emphasizes the need to better understand the natural history of *Ca*. N. procyonis. We conducted a molecular survey of raccoons from selected regions of the United States and Canada. Of 394 raccoons tested, 167 (42.4%) were confirmed to be positive for *Ca*. N. procyonis based on sequence analysis. There was spatial variation in prevalence with significantly higher prevalence (68%, 268/394) being detected in the Southeast region of the United States compared with all other regions, although a high prevalence (55.1%, 217/394) was detected in California. Lower prevalence was detected in the Midwest (3.8%, 15/394) and none of the raccoons from Canada were positive. These data suggest that *Ca*. N. procyonis is widespread in raccoon populations in the United States but there is spatial variation which may be related to vector distribution or some other factor. Although not known to infect hosts other than raccoons, neoehrlichiosis should be considered in cases of suspected ehrlichiosis in immunocompromised dogs or people that have no known etiologic agent.

## Graphical abstract

A total of 167/394 (42.4%) raccoons were positive for *Candidatus* Neoehrlichia procyonis. Spatial variation was noted with higher prevalences in the Southeast region of the United States and in California. *Ca*. N. procyonis is widespread in raccoon populations, but the spatial variation in prevalence may be related to vector distribution or another factor.

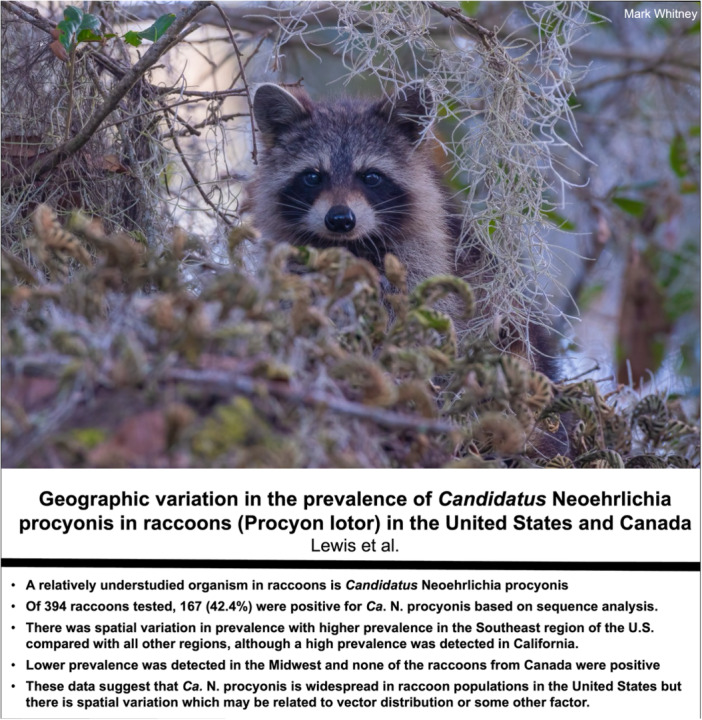

## 1 Introduction

Raccoons (*Procyon lotor*) are a highly adaptable species that are common in urban and rural habitats across much of North America where they are endemic, but raccoons have also been introduced and established in many countries in Europe and Asia (Stope 2023; Okuyama et al. 2020). Raccoons are important reservoir hosts for many pathogens of domestic animals, wildlife, and humans including rabies virus, canine distemper virus, and *Baylisascaris procyonis* (raccoon roundworm) (Ortiz et al. 2018; Cunningham et al. 2023). Raccoons are also hosts to numerous tick species and some tick‐borne pathogens throughout their native and introduced ranges (Yabsley, Murphy, Luttrell, Wilcox, et al. 2008; Yabsley, Murphy, Luttrell, Wilcox, and Ruckdeschel 2008; André 2018; White et al. 2021; Myśliwy et al. 2022; Sanjuán et al. 2022; Thompson et al. 2022; Veronesi et al. 2023). In the United States, infections of raccoons with piroplasms (*Babesia* spp.) are common (Garrett et al. 2019), but their role as hosts for Anaplasmataceae are limited. Serologic exposure to *Ehrlichia* and *Anaplasma* spp. has been reported, but only limited natural infections with *Anaplasma phagocytophilum*, *Ehrlichia canis*, and *Ehrlichia chaffeensis* have been reported (Lockhart et al. 1997; Dugan et al. 2005; Comer et al. 2000; Yabsley, Murphy, Luttrell, et al. 2008; Shultz et al. 2023). Experimentally exposed raccoons are susceptible *to E. chaffeensis, E. canis*, and *A. phagocytophilum*, but only the latter species resulted in chronic infections (Yabsley, Murphy, Luttrell, Wilcox, and Ruckdeschel 2008).

During these field studies on *Ehrlichia* and *Anaplasma* infections of raccoons, an *Ehrlichia*‐like sp. was detected and later isolated in ISE6 tick cell culture (Dugan et al. 2005; Munderloh et al. 2007). The organism was informally named *Candidatus* Neoehrlichia lotoris (Yabsley, Murphy, Luttrell, Wilcox, et al. 2008; Yabsley, Murphy, Luttrell, Wilcox, and Ruckdeschel 2008), but to follow the International Code of Nomenclature of Prokaryotes, the name *Ca*. N. procyonis was later proposed (Oren 2017). *Neoehrlichia* is a genus of obligate intracellular bacteria in the family Anaplasmataceae that contains several species. The most well‐studied species in this group is N. mikurensis which is maintained in a rodent‐tick cycle in Europe and Asia (Kawahara et al. 2004; Jahfari et al. 2012; Wennerås 2015; André 2018; Wass et al. 2019; Wang et al. 2024). Rare detections of *N. mikurensis* have been reported in mustelids, but these reports may represent *Ca*. N. sp. FU98 infections (Hofmeester et al. 2018). Known or suspected vectors for *N. mikurensis* include many *Ixodes* spp. (Kawahara et al. 2004; Wennerås 2015; Pustijanac et al. 2024). Recently, *N. mikurensis*, has been increasingly associated with clinical disease in humans and dogs (Diniz et al. 2011; Wennerås 2015; Silaghi et al. 2016; Pustijanac et al. 2024; Wennerås et al. 2024). Other *Neoehrlichia* species have at least been partially described including *Ca*. N. sp. FU98 from red fox (*Vulpes vulpes*), badger (*Meles meles*), and raccoon dogs (*Nyctereutes procyonoides*) in Europe (Hodžić et al. 2015; Hornok et al. 2017; Hildebrand et al. 2018), *Ca*. N. chilensis in rodents in Chile (Müller et al. 2018), *Ca*. N. tanzaniensis in humans from Tanzania and South Africa (Schwameis et al. 2016; Bamford et al. 2023), and two novel *Ca*. N. spp. from *Ixodes holocylus* and long‐tailed bandicoots (*Perameles nasuta*) in Australia (Gofton et al. 2015; Gofton et al. 2016; Egan et al. 2021).

To date, *Ca*. N. procyonis has only been reported from North American raccoons and there is no known vector (Yabsley, Murphy, Luttrell, Wilcox, et al. 2008, Yabsley, Murphy, Luttrell, Wilcox, and Ruckdeschel 2008, Yabsley, Murphy, Luttrell, et al. 2008). Because earlier testing of raccoons was limited to three US states (Florida, Georgia and South Carolina), the prevalence and distribution is poorly understood (Yabsley, Murphy, Luttrell, Wilcox, et al. 2008, Yabsley, Murphy, Luttrell, Wilcox, and Ruckdeschel 2008, Yabsley, Murphy, Luttrell, et al. 2008). In this study, we tested raccoons from several regions of the US and Canada to better understand the distribution of this organism in raccoons.

## 2 Methods and Materials

Extracted DNA from blood or spleen samples from raccoons from 13 US states and three Canadian provinces were obtained from other studies (Garrett et al. 2019) and clinical case submissions to the Southeastern Cooperative Wildlife Disease Study (Athens, GA) (Table 1). Extracted DNA was frozen at −20 C until testing. Molecular testing was conducted using a nested PCR targeting the 16S rRNA gene. For the primary reactions, primers ECC (5‐AGAACGAACGCTGGCGGCAAGCC) and ECB (CGTATTACCGCGGCTGCTGGCA) were used and primers GE9f (AACGGATTATTCTTTATAGCTTGCT) and GA1UR (GAGTTTGCCGGGACTTCTTCT) were used for the nested PCR as described (Dawson et al. 1994; Little et al. 1997).

**Table 1:** Prevalence of *Candidatus* Neoehrlichia procyonis in raccoons (*Procyon lotor*) from the United States and Canada.

Region and state/province	No. positive/No. tested (%)
USA Southeast	123/181 (68)
Florida	37/56 (66.1)
Georgia	54/77 (70.1)
Kentucky	1/1 (100)
Louisiana	29/44 (65.9)
Texas	2/3 (66.7)
USA Mid‐Atlantic	32/81 (39.5)
Pennsylvania	23/54 (42.6)
West Virginia	9/27 (33.3)
USA Midwest	1/26 (3.8)
Indiana	0/4
Minnesota	0/10
Missouri	1/12 (8.3)
USA Western	11/32 (34.4)
California	11/20 (55)
Colorado	0/8
Idaho	0/4
CANADA	0/74
Prince Edward Island	0/1
Nova Scotia	0/54
Ontario	0/19

Amplification products were visualized in 2% agarose gels stained with GelRed (Biotium, Hayward, CA, USA). Amplicons of appropriate size (411 bp) were purified using the QIAquick gel extraction kit (Qiagen) and submitted for bidirectional sequencing at the Georgia Genomics and Bioinformatics Core (Athens, GA, USA). Chromatograms were analyzed using Geneious R7 (Auckland, New Zealand). Fisher's Exact tests were conducted to detect if there were any differences in prevalence and state/region.

## 3 Results and Discussion

Of 394 raccoons tested, 178 had amplicons of the expected size and 167 (overall prevalence 42.4%) samples were confirmed to be *Ca*. N. procyonis based on sequence analysis (100% similar to the type specimen of *Ca*. N. procyonis, GenBank EF633744) (Table 1). The remaining sequences either failed, were mixed infections, or were other organisms such as *Serratia* spp. or *Wolbachia* spp. The *Serratia* sequence was 99.6% (232/233 bp) similar to several *Serratia* spp. (e.g., MZ311740, OR136288, PQ302163, etc.) and the *Wolbachia* sequence was a presumed endosymbiont of *Dirofilaria tenuis* and was 99.2% similar (384/387 bp) to several *Wolbachia* sequences from *Dirofilaria immitis* (e.g., CP046578. Z49261) and only 97.1% similar to *Wolbachia* endosymbionts of *Onchocerca* spp. (e.g., FR827932 and AJ276498). Novel sequences were submitted to GenBank (accession numbers PQ796469‐PQ796470).

**Figure 1:**
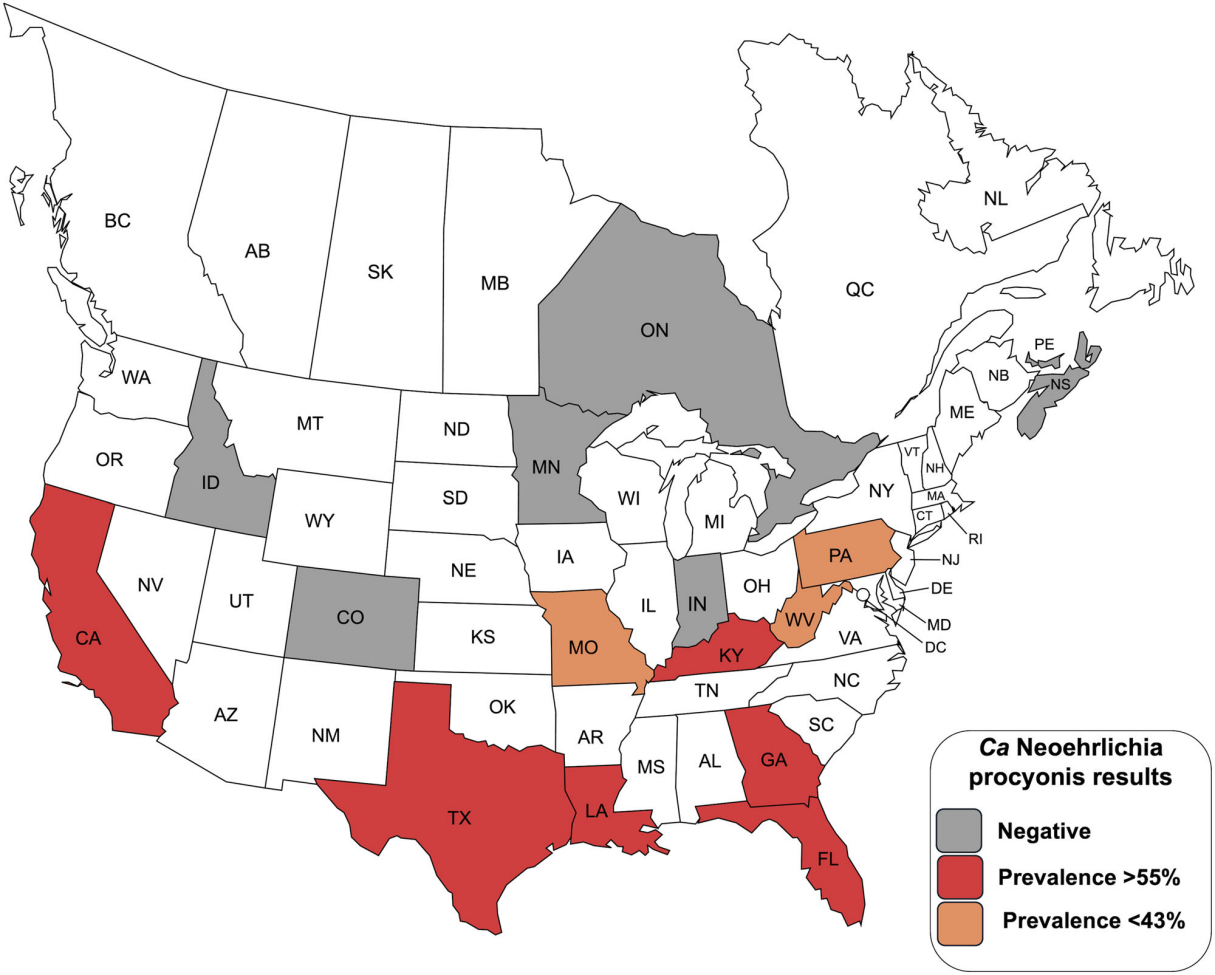
Map showing the distribution of *Candidatus* Neoehrlichia procyonis in raccoons (*Procyon lotor*) in the United States and Canada.

There was spatial variation in prevalence with significantly higher prevalence (68%) being detected in the Southeast region of the United States compared with all other regions (*p* < 0.05, Fisher's exact test) (Figure 1, Table 1). The prevalence in the Mid‐Atlantic (39.5%) and Western (34.4%) regions were also significantly higher than the Midwest and Canada regions (*p* < 0.05). At the state level, no differences were noted within regions except for the Western region where only California had positive raccoons (*p* < 0.05 between California and Colorado, the difference was not significant for California vs. Idaho). No positives were detected at the three Canadian sites.

During this study, *Ca*. N. procyonis was detected in raccoons from multiple regions of the United States; however, prevalence varied in some regions (Table 1). Raccoons in the southern and eastern areas of the United States had the highest prevalence rates, although over half of raccoons from California were positive. None of the raccoons from Canada were positive and few raccoons from the Midwest were positive. The high prevalence (68%) of *Ca*. N. procyonis we detected in the southeastern United States is similar to previous studies in Georgia, Florida, and South Carolina where > 50% of raccoons were positive (Dugan et al. 2005; Yabsley, Murphy, Luttrell, Wilcox, and Ruckdeschel 2008; Yabsley, Murphy, Luttrell, et al. 2008).

The variation in prevalence is likely due to the distribution of the vector for *Ca*. N. procyonis which is unknown. The vector is presumed to be a tick based on data from other *Neoehrlichia* spp. and because two raccoon populations previously sampled in the United States that were not infested with ticks and in two invasive populations in Europe and Asia were negative for *Ca*. N. procyonis (Yabsley, Murphy, Luttrell, Wilcox, and Ruckdeschel 2008; Sashika et al. 2011; Hildebrand et al. 2018). Previous work showed that raccoons from populations with *Ca*. N. procyonis infections were infested with *Amblyomma americanum, Amblyomma maculatum, Dermacentor variabilis, Ixodes scapularis, Ixodes texanus, and Ixodes cookei* and all of these ticks are common in the two regions with the highest prevalences (Southeast and Mid‐Atlantic region) (Yabsley, Murphy, Luttrell, Wilcox, and Ruckdeschel 2008; Nieto et al. 2018; Thompson et al. 2022). Interestingly, the presence of infected raccoons in California suggests that *I. texanus* or multiple tick species may serve as vectors because of the eastern tick species, only *I. texanus* is known to occur in California. However, vertical transmission for *N. mikurensis* has been shown to occur in rodents, so non‐tick transmission among raccoons cannot be excluded (Tołkacz et al. 2023).

The high prevalence of *Ca*. N. procyonis in raccoons, combined with the absence of disease in raccoons that are chronically infected experimentally, suggests that raccoons are the natural host. Although it appears that *Ca*. N. procyonis is specific for raccoons (Yabsley, Murphy, Luttrell, Wilcox, and Ruckdeschel 2008), neoehrlichiosis should be considered in cases of suspected ehrlichiosis in immunocompromised dogs or people that have no known etiologic agent because at least two species of *Neoehrlichia* are zoonotic. In addition, other pathogens once considered to be host‐specific for certain wildlife species (e.g., *Babesia odocoilei* of deer, *Babesia* sp. of lagomorphs) are now known to be zoonotic (Herwaldt et al. 1996; Scott et al. 2021; Maggi et al. 2024). Finally, because surveillance for this group of bacteria is predominately conducted using molecular methods, validated assays or sequence confirmation is imperative. The 16S RNA assay used in the current study was designed for the specific detection of *A. phagocytophilum*, but it is known to amplify nontarget species including *Ca*. N. procyonis, *Anaplasma odocoilei* in deer, *Anaplasma platys* in dogs, and other non‐pathogenic bacteria which can be useful for surveillance but requires sequence confirmation (Tate et al. 2013; Haynes et al. 2024; Yabsley unpublished).

## Author Contributions

**Meghan Lewis:** investigation, writing – original draft, writing – review and editing. **Kayla B. Garrett:** investigation, writing – review and editing, formal analysis, resources. **Christopher A. Cleveland:** resources, writing – review and editing. **Sonia M. Hernandez:** resources, writing – review and editing. **Mark Swain:** investigation. **Michael J. Yabsley:** conceptualization, project administration, formal analysis, writing – review and editing, writing – original draft, funding acquisition, supervision, resources.

## Ethics Statement

No animals were euthanized for the purposes of this study but the collection of biological samples for pathogen testing was reviewed and approved by the University of Georgia Institutional Animal Care and Use Committee (A2014 10‐018).

## Conflicts of Interest

None declared.

## Data Availability

Data supporting this study are included within the article.
